# Differential Diagnosis of Acquired and Hereditary Neuropathies in Children and Adolescents—Consensus-Based Practice Guidelines

**DOI:** 10.3390/children8080687

**Published:** 2021-08-09

**Authors:** Rudolf Korinthenberg, Regina Trollmann, Barbara Plecko, Georg M. Stettner, Markus Blankenburg, Joachim Weis, Benedikt Schoser, Wolfgang Müller-Felber, Nina Lochbuehler, Gabriele Hahn, Sabine Rudnik-Schöneborn

**Affiliations:** 1 Division of Neuropaediatrics and Muscular Disorders, Faculty of Medicine, University Medical Center (UMC), University of Freiburg, 79106 Freiburg, Germany; 2 Department of Pediatrics, Division of Neuropaediatrics, Friedrich-Alexander-Universität Erlangen-Nürnberg (FAU), 91054 Erlangen, Germany; regina.trollmann@uk-erlangen.de; 3 Department of Pediatrics and Adolescent Medicine, Medical University Graz, 8036 Graz, Austria; barbara.plecko@medunigraz.at; 4 Neuromuscular Center Zurich, Department of Pediatric Neurology, University Children’s Hospital Zurich, University of Zurich, 8032 Zurich, Switzerland; georg.stettner@kispi.uzh.ch; 5 Department of Pediatric Neurology, Klinikum Stuttgart, Olgahospital, 70174 Stuttgart, Germany; m.blankenburg@klinikum-stuttgart.de; 6 Institute of Neuropathology, RWTH Aachen University Hospital, 52074 Aachen, Germany; jweis@ukaachen.de; 7 Friedrich-Baur-Institute, Department of Neurology, Ludwig-Maximilians-University of Munich, Ziemssenstr. 1a, 80336 Munich, Germany; benedikt.schoser@med.uni-muenchen.de; 8 Department of Neuropaediatrics, UMC, LMU Munich, 80337 Munich, Germany; wolfgang.mueller-felber@med.uni-muenchen.de; 9 Pediatric Radiology, Institute of Radiology, Olgahospital, Klinikum Stuttgart, 70174 Stuttgart, Germany; n.lochbuehler@klinikum-stuttgart.de; 10 Department of Radiological Diagnostics, UMC, University of Dresden, 01307 Dresden, Germany; gabrie-le.hahn@uniklinikum-dresden.de; 11 Division of Human Genetics, Medical University of Innsbruck, 6020 Innsbruck, Austria; sabine.rudnik@i-med.ac.at

**Keywords:** neuropathy, children, adolescents, Charcot–Marie–Tooth disease, traumatic neuropathy, inflammatory neuropathy, metabolic neuropathy

## Abstract

Disorders of the peripheral nerves can be caused by a broad spectrum of acquired or hereditary aetiologies. The objective of these practice guidelines is to provide the reader with information about the differential diagnostic workup for a target-oriented diagnosis. Following an initiative of the German-speaking Society of Neuropaediatrics, delegates from 10 German societies dedicated to neuroscience worked in close co-operation to write this guideline. Applying the Delphi methodology, the authors carried out a formal consensus process to develop practice recommendations. These covered the important diagnostic steps both for acquired neuropathies (traumatic, infectious, inflammatory) and the spectrum of hereditary Charcot–Marie–Tooth (CMT) diseases. Some of our most important recommendations are that: (i) The indication for further diagnostics must be based on the patient’s history and clinical findings; (ii) Potential toxic neuropathy also has to be considered; (iii) For focal and regional neuropathies of unknown aetiology, nerve sonography and MRI should be performed; and (iv) For demyelinated hereditary neuropathy, genetic diagnostics should first address PMP22 gene deletion: once that has been excluded, massive parallel sequencing including an analysis of relevant CMT-genes should be performed. This article contains a short version of the guidelines. The full-length text (in German) can be found at the Website of the “Arbeitsgemeinschaft der Wissenschftlichen Medizinischen Fachgesellschaften e.V. (AWMF), Germany.

## 1. Introduction

Peripheral neuropathies are among the more frequent diseases confronting neurologists in their daily practice. The spectrum of aetiologies, clinical presentations, and disease courses is very broad, and differs considerably depending on the patient’s age. Differential diagnostics for children and adolescents can be especially challenging because the more frequent neuropathies affecting adults (the diabetic, alcoholic, and vascular forms) very seldom (if at all) affect children, whereas rare hereditary and metabolic syndromes reveal a vast aetiological spectrum. This situation is made even more difficult because electrophysiological examination methods are often distressing for children, and only a few paediatricians are trained in electrophysiological methods.

These guidelines have been drafted for a wide variety of paediatricians and specialists (neuropaediatricians, neurologists and clinical neurophysiologists, genetic counsellors and consulting services, paediatric neuroradiologists, neuropathologists, and paediatric metabolic specialists) to provide orientation regarding diseases of the peripheral nerves affecting children and adolescents. The aim of these guidelines is to describe the state-of-the-art differential diagnoses and consensus-based target- and cost-oriented diagnostics.

## 2. Materials and Methods

The methods applied for these guidelines follow the regulations of the Arbeitsgemeinschaft der Wissenschaftlichen Medizinischen Fachgesellschaften (AWMF; Version 1.1, 27.02.2013) [1]. The basis for the present version of guidelines was a non-systematic review of the recent literature by the coordinating author and by the co-operating specialists for their respective fields of expertise. All these specialists also have vast practical experience in their fields, so their clinical perspectives in addition to economic aspects have enriched these guidelines. As the Delphi technique requires, the diagnostic recommendations were considered and consented to by the whole working group in a multi-step approach. In the first step, the coordinating author collated proposals from the group members, put them into written form, and returned them to the group. Each member could then agree to a recommendation or express another opinion to be discussed. Next, the coordinating author collected all the members’ feedback and presented their discussion points in an anonymous form. Those were then sent back to the group members, who reconsidered each recommendation. This process was repeated until a solid consensus on each recommendation was achieved.

The power of a recommendation was classified in 3 levels, each with its own designation [1]:

- Strong recommendation: we recommend doing/recommend not doing
- Moderate recommendation: we suggest doing/suggest not doing
- Recommendation is open: may/can be done

The strength of consensus was classified as [1]:

- Strong consensus: >95% agreement of voters
- Consensus: >75–95% of voters
- Consent of majority: >50–75% of voters
- No consent of majority: <50% of voters

We achieved a “strong consensus” or “consensus” on all recommendations after the third round of consideration. Additionally, the text and comments of the guidelines were informally optimized by the guideline group through several voting sessions. Finally, the boards of all the participating scientific societies gave their final approval of the guidelines. The full-length text of the guidelines (in German) can be found at https://www.awmf.org/leitlinien/detail/ll/022-027.html (accessed on 6 August 2021).

## 3. Definition and Classification of Neuropathies in Children and Adolescents

Neuropathies are diseases of the peripheral and cranial nerves, whose anatomical and function-bearing structures consist of axons and myelin sheaths, endo-, peri-, and epineural connective tissue, and the vasa nervorum. Hereditary, traumatic, malignant, inflammatory, vascular, and metabolic disorders can cause damage to these structures. They can affect many nerves (polyneuropathy), or individual nerves (mononeuropathy, mononeuritis multiplex). Table 1 contains an overview.

**Nerve injuries** occur due to sharp or dull mechanical effects or tearing. A functional conduction disorder without a transected axon is termed “neurapraxia”, which heals relatively quickly. A transected axon (but with intact adjacent structures) is termed “axonotmesis”. Here, recovery is usually achieved by sprouting from the proximal axon end. In cases of “neurotmesis”, the entire nerve’s continuity is broken, frequently resulting in a scar neuroma; here, a spontaneous re-innervation is unlikely to occur [2].

**Acute para- and postinfectious neuropathies** are most frequently observed in classic peripheral facial nerve paresis (idiopathic, or infectious through Borrelia burgdorferi and varicella-zoster virus), and in a generalized form as demyelinating or axonal Guillain–Barré syndrome (GBS). These are either caused by a direct invasion by the pathogen into nerves and the spinal ganglia accompanied by inflammatory infiltrates (i.e., herpes zoster, herpes simplex, lepromatous and tubercular lepra, various parasitic agents), vasculitis disorders (borreliosis, early symptomatic HIV infection), demyelination of Schwann cells (Guillain–Barré syndrome, diphtheria), or blocking of axonal transmission by anti-ganglioside antibodies (axonal forms of GBS: acute motor axonal neuropathy (AMAN), acute motor-sensory axonal neuropathy (AMSAN)).

**Nongenetically caused metabolic polyneuropathies** can affect children and adolescents, especially in conjunction with chronic kidney failure and diabetes mellitus [3,4]. Neuropathies caused by vitamin deficiencies occur through malnutrition, resorption disorders, and insufficient parenteral feeding via a shortage of vitamin B complex (B1, -2, -6, -12) and vitamin E [5]. The cause of critical illness-neuromyopathy occasionally diagnosed in ICU patients undergoing respirator treatment is unclear [6].

**Toxic neuropathies** can be caused by medical drugs, heavy metals, organic solvents, and organic phosphoric acid esters. Their pathophysiologies are generally associated with axonal damage affecting various cellular mechanisms [5,7].

**Secondary neuropathies** in association with collagenoses or vasculitis syndromes are extremely rare in children and adolescents. They usually occur as multiplex mono-neuropathies and are pathologically characterised by segmental perivascular infiltrates and axonal lesions.

**The non-syndromic hereditary neuropathies** are clinically, genetically, pathologically, and electrophysiologically heterogeneous. The largest group among them, formerly called HMSN, is now classified according to the OMIM system and in reference to the neurologists who first described them as Charcot–Marie–Tooth (CMT) neuropathies, depending on their genetic causes. Neuropathologically speaking, they are differentiated according to what is primarily damaged—namely the axon, or myelin sheath [8]. The hereditary sensory-autonomic neuropathies (HSN) and hereditary motor neuropathies (HMN) could be regarded as CMT-related diseases with some similar features.

In addition to these isolated hereditary motor-sensory neuropathies, there is a broad range of disorders of the peripheral nerves in **complex neurometabolic and neurodegenerative diseases** revealing mainly CNS symptoms [9,10,11]. Here, usually the peripheral neuropathy manifests as a minor symptom, it is rarely the primary manifestation leading to diagnosis. Specific disease-related biochemical or histopathological findings are frequently associated with these diseases.

## 4. The Diagnostic Methods

### 4.1. History and Clinical Evaluation

**Recommendation 1:** Should a neuropathy be suspected, we recommend that a thorough patient history be taken, including the family’s history. In addition to their known medical problems, information on previous infections and toxin exposures should be acquired.

Strength of consensus: strong (10/10)

**Commentary:** The patient history should incorporate the initial symptoms and the course, and exposure to potentially causative factors in the patient’s past and family history. Early symptoms to ask for include neonatal and infantile muscular hypotonia and problems with sucking and swallowing, delayed motor development and walking, clumsiness and poor co-ordination compared to peers.

**Recommendation 2:** We recommend that a clinical examination include testing the patient’s skin and muscle trophics, their strength or paresis grade, and their reflex and sensory status.

Strength of consensus: strong (10/10)

**Commentary:** Peripheral neuropathies usually manifest as muscle weakness, loss of deep tendon reflexes, and distal muscle atrophy. Foot deformities and other contractures result from muscular imbalance and frequently manifest as pes cavus and in severe cases as equinus or club foot. Typical neurological symptoms include an inability to walk on heels, steppage gait, and abnormal co-ordination in walking and manipulation (doing buttons, peg-in-hole tests). Sensory anomalies may also appear whereby the function of the large sensory fibres (touch, deep sensitivity) is usually more strongly affected than small-fibre function (pain and temperature sensations). Sensory ataxia with a positive Romberg sign may become apparent. Autonomous skin disorders (coldness, hypohidrosis, hyperhidrosis) as well as the regulation disorders caused by impaired autonomic nerve function (i.e., bladder emptying problems) can occur.

**Recommendation 3**: We recommend that the indication for more extensive diagnostics (electrophysiology, imaging, CSF diagnostics, lab work-up, toxicology, molecular genetics, biopsy) be determined depending strongly on the patient’s medical history and clinical findings.

Strength of consensus: strong (10/10)

### 4.2. Electrophysiological Diagnostics

Clinical neurophysiology plays a key role in diagnosing neuropathies [12]. The most essential aspects to investigate when neuropathy is suspected are:

- Evidence or exclusion of peripheral nerve damage
- Identification of the affected structures (motor, sensory or sensorimotor neuropathy)
- Determination of the pathomechanism (axonal, demyelinating, or mixed damage)
- Any signs of florid denervation or re-innervation
- Signs of any additional involvement of CNS structures

It is essential that the diagnostic methods applied can be expected to yield information which extends beyond the patient’s clinical findings. To spare children from unnecessary discomfort and worry, clinical and electrophysiological diagnostics must go hand in hand, and the physician needs to have the expertise and experience to ensure this [13,14]. Electrophysiological examinations, especially needle electromyography, are considered an invasive procedure. The examiner is assumed to possess not just knowledge of the age-specific normal values [15], but also a familiarity with the methods and psychological approaches young patients require. On suspicion of a dominantly inherited CMT it can make sense to investigate the parents instead of their young child.

**Recommendation 4:** We recommend that the electrophysiological diagnostics include motor and sensory neurographies. We suggest that the exam range be oriented along the concrete issues at hand and the patient’s tolerance level.

Strength of consensus: strong (10/10)

**Commentary:** Motor and sensory neurographies help identify the neuropathies’ fundamental pathomechanism, namely whether it is primarily demyelinating or axonal (Table 2). However, with some neuropathies, it is not possible to definitively differentiate between these (mixed types, intermediate types). If a compressed nerve or other circumscribed lesion is suspected, the clinician can attempt to localise the lesion by stimulating the nerves at different anatomical points along the nerve course (latency or amplitude jump, slowing in nerve conduction velocity (NCV) in the affected section, delayed distal motor latency). Most neuropathies in childhood also reveal obvious abnormalities in sensory neurography, for example, reduced amplitude in the sensory nerve action potentials (SNAP) and possibly a slower NCV. A normal sensory neurography in a patient with purely motor symptoms should make the physician consider dHMN or DSMA.

**Recommendation 5:** We suggest that the patient undergo electromyography when seeking signs of denervation as evidence for an acute or chronic axonal neuropathy, or when there is a suspicion of an accompanying or alternative myopathy.

Strength of consensus: strong (10/10)

**Commentary:** An EMG is advisable when clarifying a neuropathy diagnosis to detect axonal lesions, i.e., for a case of acute axonal GBS, a CMT with a normal NCV, and to differentially distinguish a neuropathy from distal spinal muscle atrophy or myopathy (Table 2). This enables an assessment of pathologic spontaneous activity (positive sharp waves, fibrillations), the discharge and recruiting pattern of motor units, and the configuration of motor unit potentials (MUP). There is also an indication when seeking for signs of re-innervation, especially following traumatic neuropathy [2]. In addition, myotonic and neuromyotonic discharges can be useful in detecting diseases with muscular or nerve hyperexcitability.

**Recommendation 6:** We suggest that visual and/or acoustically evoked potentials be examined in case of a systemic disease involving the peripheral and central nervous system.

Strength of consensus: strong (10/10)

**Commentary:** The examination of visual and auditory evoked potentials enables us to assess the functionality of these specific peripheral and central sensory pathways. However, the investigation of somatosensory-evoked potentials (SEP) often fails or is inconclusive because of peripheral nerve damage. Nonetheless, with a high number of stimulations, a central summation effect can sometimes result in a recordable SEP.

### 4.3. Sensory and Vegetative Functional Diagnostics

**Recommendation 7:** Should isolated small-fibre neuropathy be suspected, quantitative sensory testing can be performed.

**Strength of consensus:** consensus (8/10)

**Commentary:** Neuropathies affecting the thin or non-myelinated (Aδ- and C-) fibres necessary for pain and temperature sensation (small-fibre neuropathy) also occur in childhood and adolescence. However, they are often detected at a late stage or not at all because of their vague or uncharacteristic clinical symptoms such as pain, fatigue, and nausea. Auto-immune disorders, adolescent-onset Morbus Fabry and familial amyloid polyneuropathy (FAP), or other hereditary sensory neuropathies are known causes [16,17]. While routine electroneurography does not usually help diagnostically, the functional disturbance can be detected via quantitative sensory testing (QST). QST has been validated for children aged 6 years and beyond, but it is currently available mainly in pain clinics [3]. However, to objectively diagnose a small-fibre neuropathy or FAP, a skin biopsy and genetic testing is ultimately required [16,17] (see also neuropathology).

**Recommendation 8:** In case of suspected autonomic neuropathy, examinations to detect an autonomic function disorder can be performed (i.e., heart rate variability, sympathetic skin reactions, and the tilt-table test).

**Strength of consensus**: strong (10/10)

**Commentary:** The autonomic nervous system should be examined, for example in case of severe GBS and hereditary autonomous neuropathies to estimate the risk of heart arrhythmias and/or cardiac arrest. This examination is available in paediatric and adult cardiology departments [18].

### 4.4. Imaging Diagnostics

**Recommendation 9:** We recommend imaging procedures (ultrasound and MRI) for cases of local or regional neuropathies without a definitive aetiological explanation; im-aging methods could reveal possible therapy-relevant lesions (nerve tumour, nerve entrapment syndrome, focal inflammation).

Strength of consensus: strong (10/10)

**Commentary:** Clinical neurological examinations are now enhanced by imaging technologies. The discovery of a causative tumour or compression enables surgery to de-compress the nerve.

**Recommendation 10:** In cases of polyneuropathies and diffuse neuropathies with doubtful clinical-electrophysiological findings and an indication to rule out an intraspinal or radicular tumour or prolapsed disc, we recommend MRI imaging or ultrasound.

Strength of consensus: strong (10/10)

**Recommendation 11:** Imaging via spinal MRI or ultrasound of the proximal nerves may also be performed to detect anomalies typical of inflammatory diseases (GBS, CIDP) in case the patient’s findings so far have been inconclusive.

Strength of consensus: strong (10/10)

**Commentary:** In patients with Guillain–Barré syndrome, CIDP, and other inflammatory radiculopathies, the spinal MRI often reveals thickening and contrast-medium enhancement in the spinal and cranial nerve roots [19,20,21]. Examinations of circumscribed and diffuse lesions and diseases of the nerve plexus and peripheral nerves via high-resolution nerve MRI or sonography are highly interesting [22,23,24]. However, as with electrophysiology, sonographic examinations of the peripheral, proximal, and cerebral nerves require a great deal of expertise and experience and are only available in few paediatric neurology and/or neuromuscular expert centres, making a general recommendation in these guidelines premature at this time.

### 4.5. Laboratory and Other Paraclinical Diagnostics

Laboratory parameters (Table 3) enable us to clarify secondary neuropathies induced by primary internal diseases (liver or kidney diseases, diabetes, collagenoses). If there are hints of a disease caused by a vitamin deficiency, the corresponding analyses should be undertaken, as in case a neurometabolic disorder in suspected [11].

### 4.6. Neuropathological Diagnostics

**Recommendation 12:** We recommend performing a nerve biopsy in case the diagnosis of a severe or progressing polyneuropathy is not otherwise possible, that is, via less invasive methods, and provided a firm diagnosis and therapy can be the consequence. This applies mainly to patients suspected of having vasculitis.

Strength of consensus: strong (10/10)

**Commentary:** Determining the indication for a nerve biopsy cannot be taken lightly; it requires great caution and differentiation. It is particularly significant for differential diagnostic workup when neuropathies are being considered that are not hereditary and can be effectively treated. In these categories belong infections such as vasculitis and peri-neuritis, as well as atypically presenting inflammatory neuropathies (chronic inflammatory demyelinating or axonal neuropathy), nerves compromised by a lymphoma, and amyloid neuropathy [25,26]. Histological tests are generally done in the sensory sural nerve and require a compression-free section of the nerve. A part thereof is fixed in formalin for paraffin histology including Congo red or thioflavin-staining and immunohistochemistry; the other is fixed in glutaraldehyde (embedding in synthetic resin for semi-thin section/toluidine blue-staining and possible electron microscopy) [27]. Nerve biopsies should only be carried out and analysed in highly specialised centres; collecting adequate tissue from infants is extremely difficult and requires a very experienced surgeon [28].

Suspected inherited sensory neuropathy or small-fibre neuropathy (SFN) calls for a **skin punch biopsy** including immunohistochemistry of epidermal and dermal nerve fibres with staining for Protein Gene Product 9.5 (PGP9.5); it also allows the study of dermal myelinated fibres, autonomic innervation (sweat glands, arrector pili muscle, arterio-venous anastomosis), and mechanoreceptors. It is a less invasive procedure used to obtain diagnosis, but it also requires expertise and the knowledge of norm values appropriate to the given age group [17,29]. Adolescent-onset FAP can be suspected by the presentation of amyloid deposits but needs genetic testing of the TTR gene for confirmation [16].

### 4.7. Genetic Diagnostics

**Recommendation** 13: In the case of suspected hereditary neuropathy we recommend molecular genetic diagnostics which include different methods depending on the patient’s clinical findings and family’s medical history.

Strength of consensus: strong (10/10)

**Comment:** Over 100 genes have been reported as being responsible for causing CMT neuropathies. The diagnostic algorithm depends on multiple factors of the presenting patient. If the family is known to carry a specific mutation, that can be verified in the patient via MLPA or Sanger sequencing. If CMT neuropathy is suspected without a known mutation, the first diagnostic step should be to identify the PMP22 gene’s copy number (usually via MLPA), especially in the case of demyelinating polyneuropathy. If that result is inconspicuous, massive parallel sequencing (next-generation sequencing (NGS)) is currently usually carried out, as it enables the most rapid and cost-effective analysis of many genes [8].

The **Gene Diagnostics Law** (GenDG) has been in effect in Germany since 2010. It mandates that special measures be taken to ensure that patients are well-informed before consenting to such tests. While any physician can schedule and carry out diagnostic examinations in patients with symptoms, the use of predictive genetic tests in healthy persons who carry a risk or in individuals with a possible genetic predisposition must be preceded by genetic counselling, which must only be conducted by certified physicians qualified to engage in genetic counselling and consultation. The genetic counselling must also include the information that molecular analysis may bring to light additional findings from genome diagnostics raising entirely different issues such as a hereditary cancer risk or risk for other neurological diseases.

## 5. Differential Diagnosis of Acquired and Hereditary Neuropathies in Children and Adolescents

### 5.1. Nerve Injuries

**Recommendation14:** If peripheral nerve injury is suspected, we recommend a clinical examination to locate the affected nerve and lesion site, and to supplement this if needed by a neurophysiological investigation.

Strength of consensus: strong (10/10)

**Commentary:** Some injured or malfunctioning nerves are clinically practically impossible to identify; they can only be objectively assessed electrophysiologically (i.e., weakness in the palmar hand muscles with a failing pre-load in radial paresis) [2].

**Recommendation 15:** We recommend a liberal indication to perform imaging (especially ultrasound) diagnostics in patients with a nerve lesion, particularly when operative treatment is likely necessary (i.e., neuronotmesis, nerve compression in a fracture gap, compression by a haematoma or tumour).

Strength of consensus: strong (10/10)

**Commentary:** Lesions affecting individual nerves or nerve plexus are often caused by typical accidents or traumas. Their clinical symptoms depend on the function of the affected nerve (motor-sensory mixed) and on the lesion’s location. An entirely transected nerve leads to paralysis of the innervated musculature, loss of sensation, and when the N. medianus and tibialis are transected, to the loss of sweat secretion in that region. Should the patient reveal some remaining function, the likelihood of continuity and spontaneous recuperation rises. Table 4 illustrates the main postnatal lesions and their motor and sensory symptoms as well as their most frequent causes.

**Recommendation16:** Electrophysiological tests can be done repeatedly to follow up patients and keep track of denervation and re-innervation processes, thus enabling a more accurate prognosis after a nerve lesion.

Strength of consensus: strong (10/10)

**Commentary:** A nerve‘s continuity can be confirmed via electrophysiological tests in patients suffering from total clinical paralysis following a nerve lesion. For patients who fail to fully recover after a nerve trauma, we suggest electrophysiological controls at 3-month intervals to evaluate the extent and direction of re-innervation, as they yield such information earlier than clinical examinations can [2].

### 5.2. Mononeuritis, Mononeuritis Multiplex

**Recommendation 17:** We recommend brain MR imaging in case of a suspected non-idiopathic facial nerve palsy; this is especially recommended in case of multiple cranial nerve lesions.

Strength of consensus: strong (10/10)

**Recommendation 18:** We recommend that infectiological blood tests be run for clinically suspected cases of borreliosis to confirm the aetiology; we suggest a lumbar puncture and cell count as well as serological tests in CSF be carried out to diagnose CNS involvement.

Strength of consensus: consensus (9/10)

**Commentary:** A peripheral or nuclear lesion of the facial nerve leads to paralysis in the mimic muscles innervated by all three of its branches. In contrast, a lesion in the corticobulbar tract leaves the function of the frontal branch intact thanks to bilateral cortical representation. Depending on where it is located, a nerve lesion along the facial canal in the base of the skull can cause lacrimal secretion to fail, as well as a loss the stapedius-reflex with hyperakusis and a loss of taste sensation on the affected side. Isolated facial paresis in childhood is usually idiopathic and inflammatory (Bell’s palsy). However, during the summer and autumn, cases of facial paresis are frequently caused by neuroborreliosis. It is often accompanied by minor symptoms of meningeal irritation; mononuclear CSF pleocytosis is detected in over 90% of such cases. The presence of pleocytosis and elevated Borrelia antibody titers in the CSF are required as evidence of neurological involvement [30]. Other causes of facial nerve palsy are zoster oticus, otitis media, petrosal bone fractures, and tumours in the brain stem and cerebello-pontine angle [31].

**Further infectious forms of neuritis** are manifested in the context of specific infections (borreliosis, zoster, diphtheria, leprosy). These can strongly determine each disease’s presentation, or go largely undetected as secondary phenomena. Their clinical symptoms are focal or multifocal, and cranial nerves are often affected. Cases of symmetric polyneuritis are seldom: in that case, it can be difficult to differentiate these from a post-infectious Guillain–Barré syndrome.

**Recommendation 19:** In cases of suspected vasculitis neuropathy, we recommend a biopsy if a firm diagnosis has proven impossible with other less invasive methods (for example, to detect typical antibodies).

Strength of consensus: strong (10/10)

**Commentary:** Cranial or spinal neuropathies or mononeuritis multiplex can appear in conjunction with different inflammatory systemic diseases. They can occur in lupus erythematodes, polyarteriitis nodosa, granulomatosis associated with polyangiitis Wegener, eosinophilic granulomatosis associated with polyangiitis Churg–Strauss, Boeck’ disease, Schönlein–Henoch syndrome, inflammatory intestinal disorders, and other autoimmune diseases [32,33]. Guillain–Barré syndrome may also be present in conjunction with these illnesses, an important factor to consider in terms of the different therapeutic consequences.

### 5.3. Guillain–Barré Syndrome (GBS) and Chronic Inflammatory Demyelinating Polyneuropathy (CIDP)

We recommend consulting the corresponding S3 guidelines for the diagnosis and treatment of acute **GBS**, whose progressive phase is limited to 4 weeks [34]. CIDP has to be assumed if a patient exhibits a longer protracted, progressing, or fluctuating disease course.

**Recommendation20:** In the case of prolonged demyelinating polyneuropathy revealing an obviously fluctuating or progressing course we recommend diagnostics for a suspected CIDP (including a CSF protein and cell count, possibly also a spinal MRI); we also recommend therapy attempts involving intravenous immunoglobulin (IvIG), plasmapheresis, or corticosteroids for inconclusive cases.

Strength of consensus: strong (10/10)

**Commentary:** CIDP can affect all age groups. Unlike acute GBS, CIDP exhibits a chronic, continuous or stepwise progressing or relapsing–remitting course. Paediatric diagnostic criteria for CIDP mandate a progressing period lasting at least 4 weeks. However, up to 20% begin as acute GBS (aCIDP) and move from there to a chronic or relapsing course [35,36]. The clinical symptoms consist of motor and sensory impairments, where the impairment of just one function is more seldom. The neuropathy is usually symmetrically distributed, primarily in the distal legs. However, the symptoms can first appear in the arms and involve the neck muscles. The cranial nerves are often affected; however, respiratory insufficiency is less frequent than in patients with GBS. Additional diagnostic criteria comprise increased CSF protein in conjunction with a normal cell count, as well as an electrophysiological proof of multiple segmentally demyelinated nerves. Infectious, toxic, or metabolic neuropathies and a CNS process entailing a distinct sensory level and a paralysed sphincter must be ruled out. A high percentage of these patients lose the ability to walk unaided. The disease can last for months or many years [35,36,37]. Potentially effective therapies for CIDP are IvIG, plasmapheresis, or prednisolone of adequate duration (i.e., at least 3 months).

**Recommendation 21:** For patients with CIDP who are resistant to treatment with IvIG, plasmapheresis, and steroids, we recommend considering and testing for potential antibodies to paranodal proteins. We also recommend the patient to be re-examined for a potential hereditary aetiology (CMT). From adolescence onwards, familial amyloid polyneuropathy has to be considered, as it is frequently misrecognized as CIDP in the beginning [16].

Strength of consensus: strong (10/10)

**Commentary:** For children presenting a therapy-resistant and protracted course of a demyelinating polyneuropathy, we propose considering the possibility of a causation through antibodies to paranodal proteins like neurofascin-155; these “paranodopathies” may respond to rituximab even when the patient is IvIG-resistant [38,39,40]. Very slowly progressing CIDP is easily mistaken for a subacute course of **hypo- or demyelinating CMT**, and vice versa [41]. Increased CSF protein and excessive contrast medium absorption in the nerve roots on a spinal MRI are typical of CIDP, but neither is specific, and both can also accompany hereditary CMT neuropathies.

### 5.4. Toxic Neuropathies

**Recommendation 22:** We recommend that, in children also, the possibility of a toxic neuropathy always be considered. This can usually be ruled out by taking a careful patient history and ensuring the child undergoes a thorough clinical-electrophysiological examination. Specific laboratory investigations are rarely needed.

Strength of consensus: strong (10/10)

**Commentary:** A partial list of potential neurotoxic substances is found in Table 5.

### 5.5. Hereditary Non-Syndromic Neuropathies in Children and Adolescents

#### 5.5.1. Hereditary Motor-Sensory Neuropathies (HMSN), Charcot–Marie–Tooth (CMT)-Neuropathy

**Clinical Presentations of CMT Neuropathies**

The clinical evidence of **classic CMT neuropathy** consists of symmetric weakness and atrophy in the distal leg muscles, weak deep tendon reflexes, and a neurogenic talipes cavus. The disease’s range of expression is extremely wide. These neuropathies usually become apparent in the **first** two decades of life. Most of their subtypes progress only slightly. Symptoms can spread to the thigh and hand musculature years later. Many of those affected exhibit few symptoms even in old age and are not detected until the family is screened. Yet, other individuals in the same family can present with early generalised weakness and suffer a very severe course.

The most frequent type of CMT is **CMT1**, the **demyelinating type.** It can be detected electrophysiologically, showing a homogeneously slowed motor nerve conduction velocity (NCV) of <38 m/s in the arm nerves (with the median nerve as reference nerve). The **axonal variant** of CMT neuropathy (**CMT2**) cannot be differentiated clinically from the demyelinating type; however, overlapping and mixed forms often make confirming a specific diagnosis very difficult. The situation is similar with **CMTX, the X-chromosomal dominant variant**, which is clinically usually apparent in males as CMT1, and in females often as axonal or mixed demyelinating-axonal neuropathy. **Additional symptoms** like hearing loss, optic atrophy, vocal cord paralysis, conspicuously rapid progression, atypical patterns in how the pareses are distributed, scoliosis, or renal insufficiency are potential signs of certain gene mutations [42,43].

**Congenital HMSN/CMT neuropathies** first becoming obvious in infancy are very rare, but nevertheless relevant for the paediatric neurologist. Because of their typical findings, these diseases used to be categorised as congenital hypomyelinating polyneuropathy and Déjerine–Sottas syndrome (previously CMT3) with demyelinating and hypertrophic neuropathy, pronounced sensory impairments, increased CSF protein, and a severe course. Molecular genetic evidence has shown that these are not independent genetic entities, but rather the clinically most severe manifestations of known phenotypically highly variable CMT mutations [44].

**Episodic neuropathies** are difficult to diagnose accurately because of their on–off phases. The most important form is autosomal-dominant **hereditary neuropathy with pressure palsies** (HNPP), with a prevalence of 7–16 individuals out of 100,000. This is characterised by recurring functional focal deficits in the peripheral nerves, especially at certain anatomically critical pressure points. It can develop over time into a chronic CMT1 or CMT2. Autosomal dominantly inherited **hereditary neuralgic amyotrophy** (HNA) is a further disease, which is characterised by burning shoulder pain and later, muscle atrophy.

**Genetic Diagnostics for CMT Neuropathies**

It is much more successful to confirm a genetic diagnosis of CMT1 (50–80% of all CMT patients) than of CMT2 (10–30% of patients). According to larger studies following comparable protocols, we can genetically identify the four most frequent genes (PMP22, GJB1/Cx32, MPZ/P0, MFN2) in 40–60% of patients in whom an inherited neuropathy is suspected [8]. In patients with CMT1A, PMP22 duplication is detected in 50–70% of cases, GJB1/Cx32 mutations in 9–18%, and MPZ/P0 mutations in 3–10%. The study results are more variable in patients with CMT2: GJB1/Cx32 in 7–19%, MFN2 in 2–20%, and MPZ/P0 in 1–6% of cases. The most frequent genes associated with autosomal-recessive CMT types are GDAP1 and SH3TC2. Mutations in GDAP1 are the most prevalent finding in autosomal dominant and recessive CMT2 cases in Spain and South Italy [45,46]. The HINT1 gene is especially prevalent in eastern Europe (Czech Republic); its clinical presentation is that of an axonal neuropathy accompanied by neuromyotony [8].

**Recommendation 23:** We recommend molecular-genetic diagnostics in case of a suspected demyelinating CMT neuropathy, starting by determining the number of PMP22 copies. This can also be done as the initial diagnostic step in case of an axonal CMT neuropathy.

Strength of consensus: consensus (9/10)

**Commentary:** Quantitative analysis of the PMP22 gene via MLPA to detect deletions and duplications is a rapid, reliable test method, while these larger duplications and deletions are not detected via NGS and Sanger sequencing. PMP22 duplications are identified in 50–70% of CMT1 patients. PMP22 deletions seldom also manifest as axonal CMT: CMT2 was clinically diagnosed in 1% of a series of 334 patients with PMP22 deletions. This PMP22 deletion, however, amounted to 5.3% of the group with genetically proven CMT2 (n = 57), making it the fourth most frequent CMT2 aetiology [47].

**Recommendation 24:** Should an HNPP be suspected, we recommend (once a PMP22 deletion has been ruled out) analyses seeking intragenic PMP22 mutations.

Strength of consensus: strong (10/10)

**Commentary:** HNPP is characterised by mainly heterozygotic deletions of the PMP22 gene; loss-of-function point mutations in the PMP22 gene are found more seldom.

**Recommendation 25:** After ruling out PMP22 duplication or deletion, we recommend performing massive parallel sequencing of gene candidates when CMT neuropathy is suspected.

Strength of consensus: strong (10/10)

**Commentary:** It is expected that advances in genetic testing will identify more and more genes in which mutations lead to ultra-rare hereditary conditions including neuropathies, and that the frequency of causative mutations will be better defined via high-throughput technologies which enable numerous genes to be examined simultaneously. The most up-to-date information on the genes associated with neuropathies is found in the Online Mendelian Inheritance in Man data base (OMIM, www.ncbi.nlm.nih.gov/omim (accessed on 6 August 2021)). The US National Institutes of Health/NIH also provide an overview of genetic diagnostics for HMSN/CMT in their gene reviews (http://www.ncbi.nlm.nih.gov/books/NBK1358/ (accessed on 6 August 2021)).

#### 5.5.2. Distal Hereditary Motor Neuropathies (dHMN)

This is a rare and genetically heterogeneous group of diseases characterised by exclusively motor symptoms affecting the distal muscle groups. Muscle atrophies and pareses primarily affect the lower extremities, but there are also forms more likely to affect the arm and hand muscles. There is some clinical and genetic overlap with distal spinal muscle atrophies that by electrophysiological and histological definition reveal no pathology in the peripheral nerves and exhibit neurogenic re-organisation in the affected musculature. dHMN can also overlap with spastic paraplegia (e.g., BSCL2). Thus, clinicians should pay attention to pyramidal signs and eventually perform motor evoked potentials.

**Recommendation 26:** We recommend performing massive parallel sequencing of gene candidates if a dHMN is suspected. PMP22 copies can be quantified beforehand.

Strength of consensus: consensus (9/10)

**Figure 1:**
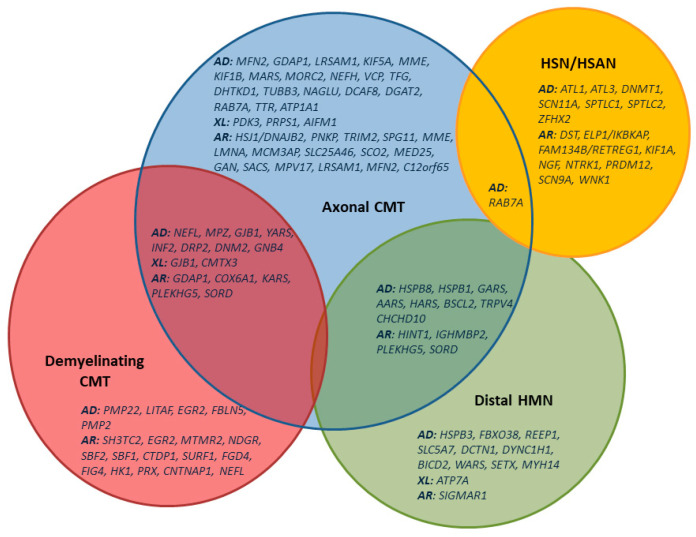
VENN diagram showing the distribution of mutations among clinical and electrophysiological groups of hereditary neuropathies (CMT/HSAN/dHMN) [8].

**Figure 2:**
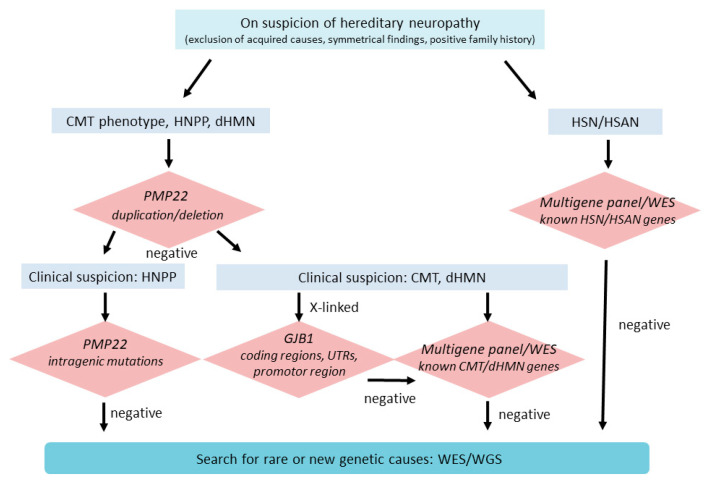
Algorithm to perform a genetic diagnosis of hereditary neuropathies [8].

**Commentary:** The genetic clarification of dHMN/DSMA has improved greatly through high-throughput technologies in the last few years, and now lies in the range of 30–40% [48]. Because of dHMN’s clinical and genetic overlapping with axonal CMT neuropathy, the genetic diagnostics are usually done simultaneously (Figure 1 and Figure 2).

#### 5.5.3. Hereditary Sensory and Autonomous Neuropathies (HSAN, HSN)

This group of extremely rare hereditary polyneuropathies is referred to as HSAN or HSN. It is primarily characterised by distally pronounced sensory-functional impairments and autonomic symptoms, and much less by minor motor impairments. Its classification in five different types becomes that much harder to follow the faster and more specifically new genes are being identified [49,50].

The autosomal-dominantly inherited **HSAN I types** usually become apparent in the second decade of life and begin with pain and impaired temperature sensation. Patients later suffer from the loss of other sensory capacities and spontaneous pain. The loss of sensory innervation triggers trophic anomalies and ulcers on the hands and feet, and not seldom gives rise to osteomyelitis and osteolysis. Less important are autonomic functional disorders (like excessive perspiration). **HSAN II–V** is inherited autosomal-recessively and usually first manifests in infancy or childhood; **HSAN II** is characterised by painless injuries (through the loss of sensation) and acrodystrophy and joint degeneration. **HSAN III** is also known as Riley–Day syndrome or familial dysautonomy; it causes autonomic-regulation impairments, vomiting, and primarily psychomotor retardation. **HSAN IV**’s dominant features are generalised anhidrosis accompanied by episodic bouts of fever already during infancy (CIPA) together with the loss of pain sensations and cognitive deficits. Children with **HSAN V** reveal no cognitive deficits, but otherwise a clinical presentation resembling that of HSAN IV [49].

**Recommendation 27:** Molecular genetic diagnostics via massive parallel sequencing of gene candidates is recommended to clarify cases of a suspected HSN/HSAN (see Figure 1).

Strength of consensus: strong (10/10)

**Commentary:** According to the very latest evidence, the responsible gene defects are only detected in 10–20% of families affected by HSAN/HSN [49,50]. As of the time these guidelines were compiled, no large investigations had been conducted targeting multi-gene panels or examination sequencing in association with this very rare group of diseases.

#### 5.5.4. Genetic Diagnostic Algorithms

To decide which genes should be analysed to clarify the aetiology of a suspected hereditary neuropathy, it is first important to clinically specify the disease as CMT neuropathy, episodic neuropathy, purely motor dHMN, purely sensory HSN/HSAN, or neuropathy in the context of a multi-system disease (Figure 2).

A quantitative procedure (MLPA) is initially recommended in the case of suspected demyelinating CMT neuropathy or HNPP to identify the frequent PMP22 duplication or deletions (Figure 2). While this diagnostic approach will fail in case of HSAN, for axonal CMT or dHMN it can be performed, although with less expectation of a positive result than in CMT1/HNPP. If the MLPA finding is negative, massive parallel sequencing of gene candidates is nowadays standard for nearly all hereditary neuropathies.

As the four most frequent genes (PMP22, GJB1/Cx32, MPZ/P0, MFN2) are detected in 90–95% of positively genetically screened patients belonging to cohorts of mainly middle-European heritage [8], clinicians are justified to first have these genes assessed after clinical and thorough formal genetic differentiation before expanding their genetic investigation to detect rare genes.

**Recommendation 28:** If an X-chromosomal CMT neuropathy is suspected, we recommend analysis of the GJB1 gene including coding regions, untranslated regions (UTRs), and promoter regions.

Strength of consensus: strong (10/10)

**Commentary:** 10–15% of patients with an X-chromosomal CMT reveal GJB1 mutations in non-coding regions that cannot be detected via high-throughput sequencing (Figure 2).

**As soon as a genetic diagnosis has been made, the affected family should be offered genetic counselling** **.**

### 5.6. Peripheral Neuropathies Associated with Complex Genetic Diseases

**Recommendation 29:** Should a systemic neurodegenerative or metabolic disorder be suspected in a patient with polyneuropathy, we recommend that primarily those diseases for which there are effective therapies be ruled out.

Strength of consensus: strong (10/10)

**Commentary:** Peripheral neuropathies can also occur in a complex array of symptoms caused by a large number of different neurometabolic and neurodegenerative syndromes. Thanks to the progress made recently in genetic diagnostics, their numbers have risen dramatically. In their systematic review, Rossor et al. [11] describe over 150 genetically defined diseases. Only a small proportion of these are manifested in childhood, and many have only been described in one or a handful of families. The neuropathy is often just one aspect of a given disease, although it can cause the main symptoms for some time during the disease’s initial stage. For a given genetic disease, the neuropathy may present as an isolated symptom in adulthood if the mutation’s effect is weak; but children whose gene is largely dysfunctional usually suffer from progressive CNS deterioration and early death. It is significant to note that effective therapies are available for some of these diseases provided they are diagnosed early enough, which is why they must not be diagnostically overlooked (Table 6).

A list of relevant systemic diseases in childhood is found in Table 7. The format of these guidelines did not permit the provision of more details. For more information, please see [9,10,11].

## 6. Conclusions

Diseases of the peripheral nerves with highly diverse aetiologies affect children and adolescents as well as adults. The spectrum of their causes in children differs strongly from that of adults. The differential diagnostic approach first requires fundamentally solid anatomical and neurophysiological knowledge and understanding, with thorough analyses of the disease course and family history, the disease’s topographic distribution, and the quality of the patient’s neurological symptoms. Supplemental examinations such as electrophysiology, laboratory workups, imaging, and CSF diagnostics follow a clinically driven hypothesis. That also applies to molecular genetic diagnostics, which require experience with and a command of the diagnostic algorithms due to the enormous genetic heterogeneity of hereditary neuropathies. These again will be expanded as the potential of broad-based panel or exome diagnostics is realized. To ensure effective and well-targeted therapy, a diagnosis as precise as possible is decisive—not just to alleviate nerve injuries, but also for patients with inflammatory and metabolic/malnutritional neuropathies. The causes of genetic neuropathies should not just be investigated in preparation for genetic counselling. Such knowledge is essential to be able to inform the patient about the disease’s probable course and prognosis, and to enable them to participate in present and future clinical trials addressing pathologies and therapies.

## Figures and Tables

**Table 1:** Survey of neuropathies in children and adolescents.

Hereditary Neuropathies	Acquired Neuropathies
**1** **.** **Hereditary Motor-Sensory Neuropathies (HMSN)/Charcot–Marie–Tooth (CMT) Neuropathies** CMT1 (demyelinating, AD) CMT2 (axonal, AD or AR) Intermediate CMT (AD or AR) CMT4 (demyelinating, AR) CMTX (demyelinating, axonal, XD, XR) **2** **.** **Hereditary Sensory-Autonomous Neuropathies (HSAN)** HSAN1(AD) HSAN2-8 (AR) **3** **.** **Hereditary Motor Neuropathies (HMN)** HMN (AD) DSMA (AR) DSMAX (XD) **4** **.** **Episodic Neuropathies** HNPP (AD) HNA (AD) **5** **.** **Syndromic neuropathies** Neuropathies in the context of neurometabolic diseases Neuropathies in the context of neurodegenerative diseases	**1** **.** **Polyneuropathies** Inflammatory (infectious, postinfectious (GBS, CIDP), vasculitis) Toxic (drugs, heavy metals) Metabolic/malnutrition (uremia, diabetes, dysproteinemia, vitamin deficiency, hypervitaminoses) Paraneoplastic Critical-illness neuropathy Small-fibre neuropathy **2** **.** **Mononeuritis multiplex** Inflammatory **3** **.** **Mononeuropathies** Inflammatory Traumatic Nerve tumours Nerve compression syndromes **4** **.** **Neuralgic shoulder amyotrophy, plexopathies** Traumatic Inflammatory

DSMA: distal spinal muscular atrophy; HNPP: hereditary neuropathy with pressure palsies; HNA: hereditary neuralgic amyotrophy; AD: autosomal dominant; AR: autosomal recessive; XD: X-chromosomal-dominant; XR: X-chromosomal recessive. Comprehensive names for groups of diseases are printed in bold.

**Table 2:** Electrophysiological criteria for demyelinating or axonal damage to peripheral nerves.

	Motor Neurography	EMG
Demyelination	Slowing of NCV, prolongation of distal-motor latency, prolongated or deficient F-waves (in proximal demyelination), abnormal dispersion of CMAP and partial conduction block (acquired multifocal demyelination, for example CIDP)	Normal findings when axonal anomalies are absent
Axonal damage	Lowered CMAP amplitude (can also be due to muscular atrophy/myopathy)	Florid denervation: fibrillation potentials, positive sharp waves, reduced interference pattern Chronic denervation with re-innervation: abnormal high-amplitude and polyphasic potentials, reduced interference pattern

NCV: nerve conduction velocity; CMAP: compound muscle action potential.

**Table 3:** Laboratory tests for clinically and electrophysiologically suspicious cases.

Disorder	Lab Diagnostics
Infectious neuropathy	Serology and possible swabs, *Borrelia burgdorferi*, VZV, etc.
Inflammatory systemic diseases	ESR, CRP, ANA, double-stranded DNS-ab, C3 complement, ACE, lysozymes, immunoelectrophoretis, cryoglobulin
GBS, CIDP	CSF protein, CSF cell count, pathogen serology (CMV, *Mycopl. pneumoniae*, *Campylobacter jejuni*), anti-gangliosid antibodies (seldom positive), antibodies against paranodal proteins (neurofascin, etc.)
M. Refsum and other peroxisomopathies	Phytanic acid, VLCFA in serum
Bassen–Kornzweig syndrome (a-betalipoproteinemia)	Electrophoresis, lipid electrophoresis, vitamin E
Primary vitamin E resorption disorder	Vitamin E
Secondary vitamin resorption disorders	Vitamin D (Ca, P, AP, parathormone), vitamin E, vitamin K (INR, quick value), folic acid, vitamin B12 (with holotranscobalamin and methylmalonic acid), thiamin, riboflavin. Vitamin B6 (overdose?)
CDG syndromes	Isoelectric transferrin focussing
Metachromatic leukodystrophy and M. Krabbe	CSF protein, lysosomal enzymes
Mitochondriopathies	Lactate in plasma and CSF, possible muscle biopsy for biochemical analyses

**Table 4:** Clinical appearance and aetiology of peripheral nerve lesions.

Site of Lesion	Motor Defect	Sensory Defect	Aetiologies
Upper brachial plexus	Shoulder abduction and external rotation, elbow flexion, supination	Lateral/radial arm from shoulder to metacarpo-phalangeal joint of thumb	Strain/tearing of the shoulder, perinatal trauma, neuralgic amyotrophy, serogenetic neuritis, tumour infiltration
Lower brachial plexus	Finger and wrist flexion, finger ab- and -adduction, possibly Horner syndrome	Axillary and ulnar side of the arm from elbow to hand and finger IV and V	Trauma as above, abnormal cervical rib, scalenus syndrome, tumour infiltration
Long thoracic nerve	Elevation and rotation of scapula, winged scapula on shoulder flexion	-	»back packer palsy«, neuralgic amyotrophy
Radial nerve	Extension of wrist and metacarpo-phalangeal joints, abduction of thumb, extension of thumb and finger II (»flaccid drop of the hand«)	Back of the hand overlying metarcapals I and II	Humerus shaft fracture, pressure palsy
Median nerve	Flexion of wrist, flexion of finger I–III (»Schwurhand«)	Volar side of the hand and fingers I to the radial side of IV, dorsal side of same fingers	Supracondylar fracture of humerus, pressure palsy, carpal tunnel syndrome
Ulnar nerve	Flexion of wrist and metacarpo-phalangeal joint IV–V, ab-/adduction III–V, thumb adduction (»Krallenhand«)	Volar and dorsal side of hand and fingers IV and V (not radial side finger IV)	Supracondylar fracture of humerus, elbow fracture, pressure palsy
Ischiadic nerve	Combination of tibial- and peroneal lesion	Combination of tibial- and peroneal lesion	Ischiadic lesion at misplaced injection, pelvic fractures
Tibial nerve	Foot- and toe flexors, loss of Achilles tendon reflex	Plantar side of the foot, lateral side of the foot	Fractures, injury to the hollow of the knee
Peroneal nerve	Foot extension (»Steppage gait«)	Lateral lower leg, back of the foot	Fibular fracture, pressure palsy

**Table 5:** Toxic agents associated with polyneuropathy (selection).

Drugs	Heavy Metals and Solvents
Vincristin, cis-platinum	Lead
Taxane, epothilone	Gold
Bortezomib	Thallium
Thalidomide	Arsenic
Nitrofurantoin, isoniazid (INH)	Mercury
Hydantoine	n-Hexane
Chloramphenicol, metronidazol	Methyl-n-butylketone
Amphotericin	Triorthocresylphosphate

**Table 6:** Neuropathies in the context of complex hereditary diseases amenable to treatment [11].

Disease	Diagnostics	Treatment
Refsum syndrome	Axonal or demyelinated NP, phytanic acid, pristanic acid	Phytanic acid-restricted diet, plasmapheresis
Adrenoleukodystrophy	VLCFA	Presymptomatic BMT
Metachromatic leukodystrophy	Demyelinated NP, lysosomal enzymes	Presymptomatic BMT
Vitamin E malabsorption	Vitamin E in serum	Vitamin E
Bassen–Kornzweig syndrome	Abetalipoproteinemia	Vitamin E
B12 deficiencies in resorption and utilisation	Vit B12 in serum, methylmalonic acis, homocystein in serum	Vitamin B12
Folate deficiencies in resorption and utilisation	Folate, 5-Methyltetrahydrofolate	Folate, folinic acid
Cerebrotendinous xanthomatosis	Plasma cholestanol	Diet, chenodesoxycholic acid
Brown–Vialetto–van Laere syndrome	Bulbar paralysis, deafness, riboflavin in serum	Riboflavin
CD59 mutation	Anemia, paroxysmal nocturnal hematuria, relapsing NP	Eculizumab
Acute intermittent porphyria	Porphobilinogen	Glucose, haematin
Pyruvate dehydrogenase deficiency	Lactate, enzyme diagnostics, genetics	Ketogenic diet
Morbus Fabry (alpha-galactosidase A deficiency)	Severe acroparesthesias, autonomous neuropathy, angiokeratoma, corneal dystrophy, cardiovascular disease, stroke, renal impairment	Enzyme replacement therapy with agalsidase alfa or agalsidase beta
Familial amyloid polyneuropathy (onset from adolescence)	Symmetric sensory-motor and autonomic neuropathy, family history of neuropathy and/or cardiomyopathy, gi involvement, weight loss, carpal tunnel syndrome, TTR genetics	Liver transplantation, TTR stabilizers and gene modifying approaches in preparation [16]

BMT: bone marrow transplantation; VLCFA: very long-chain fatty acids; gi: gastrointestinal.

**Table 7:** Polyneuropathies in systemic neurological diseases with onset in childhood or adolescence.

Initially Presenting with PNP	+Ataxia	+Spasticity	+Ataxia +Spasticity +EPMS	+Global Developmental Delay	Multisystem Involvement
Metachromatic Leukodystrophy (*ARSA*)	Vitamin E deficiency (*TTPA, MTP, acquired*) * Vitamin B12 deficiency * Refsum syndrome (*PHYA*) * Sensory PNP in Friedreich Ataxia (*FXN*) EAOH (*APTX*) SCAR1(*SETX*) SCA27 (*FGF14*) AT (*ATM*) NARP (*MTATP6*) SCAR23 (*PDYN*) Microcephaly, seizures, and developmental delay (*MCSZ*) ARSACS (*SACS*) SCAN1 (*TDP1*)	Adrenoleukodystrophy (*ABCD1*) * Methylmalonic aciduria Vit. B12 deficiency * SPG (4, 9a, 12, 17, 39 ...)	Spastic Aataxia 5 (*AFG3L2*) LBSL (*DARS2*) Hypomyelinating leukodystrophy (TUBB4A) Leigh (-like) (SURF1/MFF, SUCLA2)	PDHC (*PDHAl*) * Krabbe (*GALC*) * Metachrom. Leukodystrophy (*ARSA*) * Aicardi-Goutieres syndrome * Global insensitivity to pain (*CTLC1*) Giant axonal neuropathy (*GAN*) NBIA2a (*PLA2G6*) CDG (*NGLYl*) MCSZ (*PNKP*) CEDNIK (*SNAP29*) Ponto-cerebellar hypoplasia (*EXOSC3/AMPD2*) Infantile Refsum (*PEX7*)	Mitochondriopathy * multi. acyl CoADH deficiency * Hexosaminidase A/B deficiency (*HEXA/HEXB*) * Brown–Vialetto–Van Laere (*SLC52A2/3*) * Peroxisomal 6 (*PEX10*) ACPHD (*ABHD12*) PHARC SPOE NBIA (*C1901f12*) SCAR21 (*SCYLl*) Familial Dystautonomia (*TECPR2*) Triple A (*AAAS*) MEDNIK (*AP151*) PTRH2 Galaktosialidosis (*CTSA*)

EAOH: early-onset ataxia, with oculomotor apraxia and hypoalbuminemia; SCAR: spinocerebellar ataxia, recessive; SCA: spinocerebellar ataxia; AT: ataxia teleangiectasia; NARP: neuropathy, ataxia, and retinitis pigmentosa; ARSACS: autosomal recessive spastic ataxia of Charlevoix–Saguenay; SCAN: cerebellar ataxia and sensory-motor axonal neuropathy; SPG: spastic paraplegia; LBSL: leukoencephalopathy with brainstem and spinal cord involvement and lactate elevation; NBIA: neurodegeneration with brain iron accumulation; CDG: carbohydrate deficient glycoprotein disorder; CEDNIK: cerebral dysgenesis, neuropathy, ichthyosis, and palmoplantar keratoderma syndrome; ACPHD: ataxia, combined cerebellar and peripheral, with hearing loss and diabetes mellitus; PHARC: polyneuropathy, hearing loss, ataxia, retinitis pigmentosa, and cataract; MEDNIK: mental retardation, enteropathy, deafness, peripheral neuropathy, ichthyosis, and keratoderma; PTRH: infantile-onset multisystem neurological, endocrine, and pancreatic disease; * amenable to treatment.

## Data Availability

Not applicable.
